# Supplementary material for: The hourglass model of evolutionary conservation during embryogenesis extends to developmental enhancers with signatures of positive selection
Source: Genome Res. 2021 Sep;31(9):1573–81. doi: 10.1101/gr.275212.121 (PMC8415374; doi:10.1101/gr.275212.121)
Supplement: Supplemental Material [file supp_31_9_1573__DC1.html]

The hourglass model of evolutionary conservation during embryogenesis extends to developmental enhancers with signatures of positive selection — Supplemental Material 

# The hourglass model of evolutionary conservation during embryogenesis extends to developmental enhancers with signatures of positive selection

## Supplemental Material

- Supplemental\_Code.zip
- Supplemental\_Figures.pdf
